# Leveraging advances in diabetes technologies in primary care: a narrative review

**DOI:** 10.1080/07853890.2021.1931427

**Published:** 2021-06-29

**Authors:** Bruce Bode, Aaron King, David Russell-Jones, Liana K. Billings

**Affiliations:** a Atlanta Diabetes Associates, Atlanta, GA, USA; b HealthTexas at Stone Oak, San Antonio, TX, USA; c Royal Surrey County Hospital, Guildford, UK; d NorthShore University HealthSystem/University of Chicago Pritzker School of Medicine, Skokie, IL, USA

**Keywords:** Continuous glucose monitoring, diabetes technologies, insulin delivery systems, self-measured blood glucose, time in range

## Abstract

Primary care providers (PCPs) play an important role in providing medical care for patients with type 2 diabetes. Advancements in diabetes technologies can assist PCPs in providing personalised care that addresses each patient’s individual needs. Diabetes technologies fall into two major categories: devices for glycaemic self-monitoring and insulin delivery systems. Monitoring technologies encompass self-measured blood glucose (SMBG), where blood glucose is intermittently measured by a finger prick blood sample, and continuous glucose monitoring (CGM) devices, which use an interstitial sensor and are capable of giving real-time information. Studies show people using real-time CGM have better glucose control compared to SMBG. CGM allows for new parameters including time in range (the time spent within the desired target glucose range), which is an increasingly relevant real-time metric of glycaemic control. Insulin pens have increased the ease of administration of insulin and connected pens that can calculate and capture data on dosing are becoming available. There are a number of websites, software programs, and applications that can help PCPs and patients to integrate diabetes technology into their diabetes management schedules. In this article, we summarise these technologies and provide practical information to inform PCPs about utility in their clinical practice. The guiding principle is that use of technology should be individualised based on a patient’s needs, desires, and availability of devices. Diabetes technology can help patients improve their clinical outcomes and achieve the quality of life they desire by decreasing disease burden.

**KEY MESSAGES**
- It is important to understand the role that diabetes technologies can play in primary care to help deliver high-quality care, taking into account patient and community resources. Diabetes technologies fall into two major categories: devices for glycaemic self-monitoring and insulin delivery systems. Modern self-measured blood glucose devices are simple to use and can help guide decision making for self-management plans to improve clinical outcomes, but cannot provide “live” data and may under- or overestimate blood glucose; patients’ monitoring technique and compliance should be reviewed regularly. Importantly, before a patient is provided with monitoring technology, they must receive suitably structured education in its use and interpretation.
- Continuous glucose monitoring (CGM) is now standard of care for people with type 1 diabetes and people with type 2 diabetes on meal-time (prandial) insulin. Real-time CGM can tell both the patient and the healthcare provider when glucose is in the normal range, and when they are experiencing hyper- or hypoglycaemia. Using CGM data, changes in lifestyle, eating habits, and medications, including insulin, can help the patient to stay in a normal glycaemic range (70–180 mg/dL). Real-time CGM allows for creation of an ambulatory glucose profile and monitoring of time in range (the time spent within target blood glucose of 70–180 mg/dL), which ideally should be at least 70%; avoiding time above range (>180 mg/dL) is associated with reduced diabetes complications and avoiding time below range (<70 mg/dL) will prevent hypoglycaemia. Insulin pens are simpler to use than syringes, and connected pens capture information on insulin dose and injection timing.
- There are a number of websites, software programs and applications that can help primary care providers and patients to integrate diabetes technology into their diabetes management schedules. The guiding principle is that use of technology should be individualised based on a patient’s needs, desires, skill level, and availability of devices.

## Introduction

Primary care providers (PCPs) play an ever-increasing role in the management of patients with type 2 diabetes (T2D), especially since the increasing prevalence of T2D in the general community cannot be managed solely by specialist endocrinology services [1]. As the first line of healthcare, PCPs are also well positioned to manage patients’ general health and lifestyle. Technologies can enhance the delivery of coordinated, team-based care (including specialist diabetes practitioners and educators) and promote patient self-management, which are critical aspects in the Chronic Care Model advocated by the American Diabetes Association (ADA) for tailoring diabetes care to the needs of each patient [2]. This model has been shown to reduce the incidence of diabetes-related complications and all-cause mortality [2]. Patient self-monitoring of blood glucose is also advocated by European guidelines [3]. It is important to note that the use of technology should be individualised based on a patient’s needs, desires, skill level, and availability of devices [4].

Glycaemic control is a key goal for people with T2D [5]. Many patients struggle to meet their glycaemic targets or have suboptimal glycaemic variability, putting them at risk of short- and long-term complications [5,6]. The importance of good glucose control has been recently highlighted by the presence of diabetes as a risk factor for complications and death caused by the COVID-19 virus [7]. PCPs play a very important role in monitoring and supporting patients to manage their condition and achieve/maintain glycaemic targets. Understanding diabetes technologies can also help the patient contribute to their own high-quality care [2,8]. Advancements in diabetes technology are playing an increasing role in the management of diabetes [2,8], and fall into two major categories: devices for glycaemic self-monitoring and insulin delivery systems [4,8].

Two types of devices for self-monitoring are available: self-measured blood glucose (SMBG) meters and continuous glucose monitoring (CGM) meters. SMBG meters are suitable for self-monitoring blood glucose at specific time points, whereas CGM meters measure glucose levels continuously [9]. CGM is recommended for all people with diabetes taking intensive insulin regimens [8]. Importantly, before a patient is provided with monitoring technology, they must receive suitably structured education in its use and interpretation.

In patients with T2D, insulin is usually administered by syringe or – nowadays much more commonly – by an injection pen [10]. Connected (“smart”) pens provide the potential for further advantages in monitoring, calculating the insulin dosage, and compliance [11]. Automated insulin delivery devices include insulin pumps with continuous subcutaneous insulin infusion (CSII) and closed-loop systems (sensor-augmented pumps, integrated with a real-time CGM), but these are predominantly used by people with type 1 diabetes (T1D), who are generally managed by endocrinologists rather than PCPs [4,12]. However, they may become more prevalent in the T2D domain as they become simpler to use.

In this review, we provide an overview of key diabetes technologies for PCPs and summarise current guidelines. Practical considerations on how to manage the application of these technologies in primary care (including benefits and potential pitfalls) are discussed.

## Materials and methods

To support this article, the PubMed database was searched in a non-systematic manner for relevant publications. Retrieved articles were filtered to remove duplicates and irrelevant results. The reference lists of the selected articles were checked for any other publications pertinent to this manuscript.

## Results: digital technologies for patients with diabetes

### Technology for glucose self-monitoring

#### Self-monitored blood glucose

Advantages and disadvantages of SMBG are summarised in Table 1. Patients can use an SMBG device to measure their blood glucose at specific (patient-initiated) timepoints, thus providing intermittent data [18], and have been used for decades [17]. Current devices measure glucose in a blood sample from a finger prick; glucose in blood reacts with an enzyme (glucose oxidase or glucose dehydrogenase) on a test strip to generate electrons that are detected by a sensor, which provides a digital readout of blood glucose level [18]. Modern SMBG devices are simple to use, accurate, provide rapid results, and require small blood volumes [13,16]. Performing structured SMBG helps guide decision making for clinical and self-management plans to improve outcomes [23]. However, SMBG cannot provide “live” data.

**Table 1.** Attributes of diabetes technologies approved for glucose self-monitoring [4,8,9,13–22].

	SMBG	Intermittent (Flash) CGM	Real-time CGM
	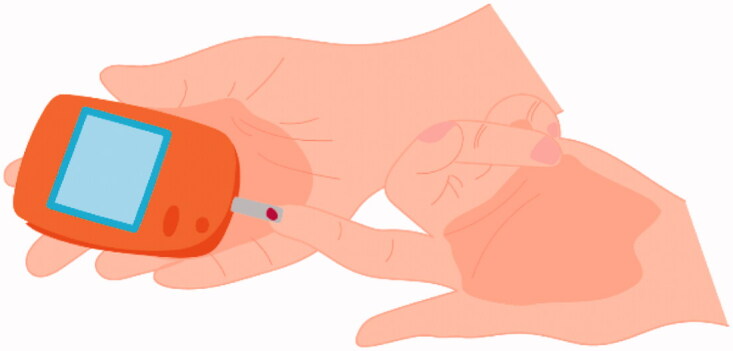	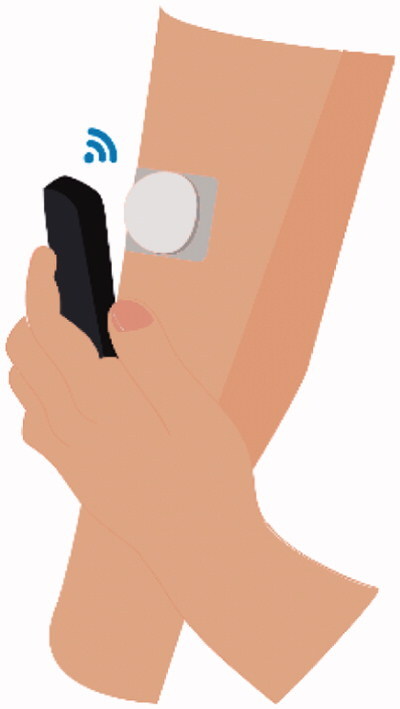	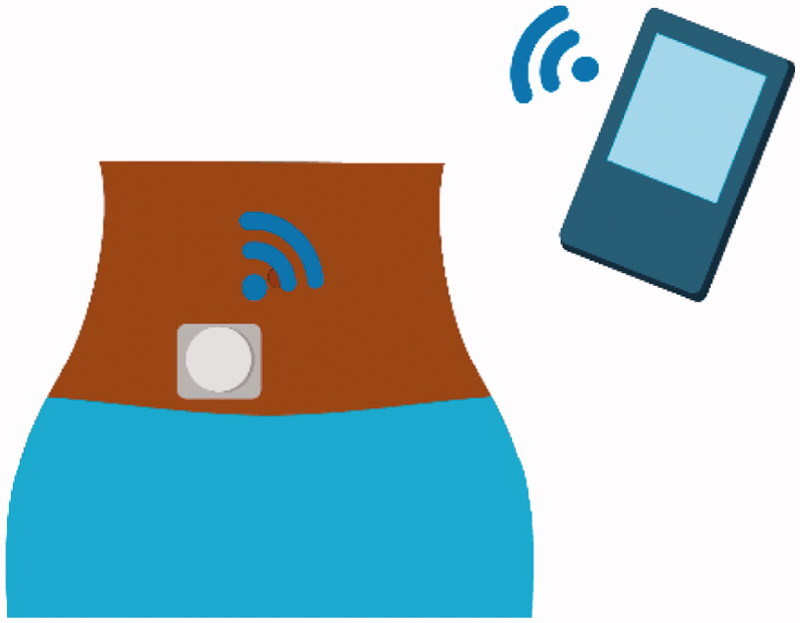
Example devices	Multiple products available	Freestyle Libre^®^	Dexcom^®^ G6, Medtronic^® ^Guardian Connect System, Eversense^®^ CGM System*
Technology	Electrochemical test strips: glucose in whole blood reacts with enzyme on strip to generate electrons detected by sensor Digital display Most have software to allow download of data from the glucometer	Sensor (with transmitter) applied to skin with small electrochemical probe sticking into tissue below Sensor measures glucose in the interstitial tissue and sends data *via* Bluetooth connection to a wireless receiver Intermittent glucose readings: when sensor swiped by a reader/smart phone	Sensor (with transmitter) applied to skin with small electrochemical probe sticking under the skin into the tissue below Sensor measures glucose in the interstitial tissue and sends data *via* Bluetooth connection to a wireless receiver Real-time continuous measurement of glucose levels; have automated alarms/alerts
Sample type	Capillary blood	Interstitial fluid (minimally invasive)	Interstitial fluid (minimally invasive)
Software and applications	Internal meter memory store between 400 and 1000 test results with date and time Free proprietary software to connect meter to a computer; generate reports Most manufacturers provide apps and/or websites for integrating data across diabetes care	Cloud-based software available to capture data, create reports and share with healthcare team (encrypted data) Mobile medical app available: glucose monitoring from smart phone (no need for separate glucose monitor) Most manufacturers provide apps and/or websites for integrating data across diabetes care	Mobile medical apps allow glucose monitoring from smart phone (avoids the need for a separate glucose monitor) Some systems allow real-time shareable data Most manufacturers provide apps and/or websites for integrating data across diabetes care
Advantages	Well established Accurate/sensitive Fast results Familiarity Easy to use and train patients Relatively cheap Confirms accuracy of interstitial glucose measurements	Interstitial glucose correlates well with blood glucose Most sensors are factory calibrated (no need for SMBG) Patients are empowered to check glucose level when required Able to capture and display trend data over time (increasing or decreasing glucose) Data saved for reviewing longer-term trends (supports ambulatory glucose profile) More affordable alternative to real-time CGM (covered by Medicare) Longer sensor life than most real-time CGM devices Builds awareness of how food choices affect blood glucose	Interstitial glucose correlates well with blood glucose Real-time data with predictive algorithms to allow attenuation of glucose highs/lows Alarms for high and low glucose values Creates ambulatory glucose profile (glycaemic variability, time in target range, etc.); supports timely treatment modifications/intensification Long-term benefits (HbA_1c_ and hypoglycaemia reductions) Smart phones can be used for capturing data (convenient, cost saving) Some CGM devices transmit data to insulin delivery devices Builds awareness of how food choices affect blood glucose
Disadvantages	Invasive sampling (inconvenient, time consuming, painful) Limited data Cannot predict impending hypoglycaemia or alert for hypoglycaemia Frequent testing is difficult to maintain long term Device-to-device and strip-to-strip variations in accuracy	Data captured limited by patient needing to remember to scan Cost of replacing sensors (last for 2 weeks) Contact dermatitis is a risk	Relatively expensive versus SMBG (sensors last for 7–14 days) SMBG required for calibration or confirmation of unexpected readings with many devices Complex; patients need guidance on what data mean and actions needed Potential for psychosocial impact (information overload, anxiety) Contact dermatitis is a risk
Other considerations	Performing SMBG alone does not lower blood glucose levels; information must guide actions for clinical and self-management plans Structured SMBG plan achieves best outcomes	Structured plan for scanning glucose levels achieves best outcomes Helps bridge the gap for people with T2D on insulin who need more intensive glucose monitoring than SMBG Cost is a consideration if no insurance coverage	Structured education required for both HCPs and people with diabetes to derive benefits of CGM Ability of a person with diabetes to cope with the data and derive benefit from CGM Cost is a major consideration if no insurance coverage

*Uses an implantable sensor rather than a probe. Additional information from product websites.

CGM: continuous glucose monitoring; HbA_1c_: glycated haemoglobin; HCP: healthcare professional; RCT: randomised controlled trial; SMBG: self-monitored blood glucose; T1D: type 1 diabetes; T2D: type 2 diabetes.

##### Practical information for PCPs

Although training patients to use an SMBG meter is straightforward, compliance with a glucose-monitoring regimen using SMBG can decrease over time, and the accuracy of readings depends on the instrument and the user. Therefore, patients’ monitoring technique should be reviewed regularly [4]. SMBG devices are battery operated and spare batteries should be carried with the person at all times. Good manual dexterity and visual acuity are needed to operate most glucose monitors. Taking the required blood sample can sometimes cause discomfort. PCPs should be aware that gaps exist between SMBG device readings and laboratory venous blood glucose levels, but a long-term survey showed differences were clinically relevant in only 1% of cases [17]. For reference, the FDA requires that SMBG devices must give 95% of all readings within ±15% of comparator results, and 99% of all readings within ±20% of the comparator, across the entire claimed measuring range. Almost all glucose meters provide data that can be downloaded to a software system to give the average number of glucose tests per day, as well as overall average and standard deviation glucose readings, and average reading at each meal and bedtime.

#### Continuous glucose monitoring

CGM devices consist of a sensor, transmitter, and wireless receiver [18]. The device is attached to the skin by means of an adhesive. A small wire-based sensor is inserted just under the skin to measure glucose levels in interstitial fluid [19,20]. The sensor measures a current generated by the glucose-oxidase reaction in the same way as the test strip in the SMBG device [18,19]. Data from the sensor are wirelessly transmitted to a receiver or smartphone app that can display real-time glucose level [21]. Two types of CGM devices exist: real-time devices that continuously capture glucose levels, and intermittent devices that capture the glucose level when a receiver is placed near the sensor. Advantages and disadvantages of each approach are summarised in Table 1.

CGM devices were introduced in 1999 [20] and allow continuous monitoring of blood glucose to provide a clear picture of a patient’s daily glycaemic profile [9]. This can result in improved glycaemic control with less time spent outside of target, ultimately reducing the risk of hyperglycaemia, hypoglycaemia and diabetes-related complications [21]. CGM may particularly benefit patients with hypoglycaemic unawareness and those at high risk of hypoglycaemia, and identify times of increased hypoglycaemic risk (e.g. at night), especially in risk groups such as the elderly and children [4]. When used appropriately, CGM can lead to average glucose concentrations that are closer to normal, reduce the severity and worry of hypoglycaemic events, and help reduce the cost of complications [22]. CGM data can be displayed in the form of the ambulatory glucose profile (AGP), which is a standardised tool for summarising large amounts of CGM data to foster discussion between the healthcare provider and the patient about patterns that warrant therapeutic adjustment [14,22]. Although CGM addresses many of the limitations inherent in SMBG monitoring, there is a need for standardised software for visualisation and reporting of key CGM metrics to support AGP [24]. Recently, an international expert panel provided recommendations to standardise CGM reporting [25].

##### Intermittent CGM (flash glucose monitoring)

Intermittently scanned CGM devices provide a snapshot of blood glucose levels at a moment in time, and also record trends in glucose levels over time. There are relatively little data from randomised controlled trials to prove a benefit, and such data are from people with T1D [4]. One study showed a significant improvement in the time spent in a hypoglycaemic range in patients with T1D [26], while other observational studies showed benefits in terms of glycated haemoglobin (HbA_1c_) reduction [15]. Use of intermittent CGM devices can be supplemented by use of a professional CGM system that can be provided to the patient for a period of up to 14 days for visualisation of AGP [1]. Intermittently scanned CGM can be more cost effective than real-time CGM and is considered useful for adults and children >4 years with T2D who are receiving insulin therapy but not meeting glycaemic targets [4].

##### Real-time CGM

There is a strong evidence base showing the benefits of real-time CGM in people with diabetes receiving insulin, including the DIAMOND T1D/T2D and GOLD T1D studies [27–32]. In both the DIAMOND and GOLD studies, HbA_1c_ levels were significantly reduced with real-time CGM versus SMBG [31,32]. In the T1D cohort of DIAMOND, reductions in HbA_1c _were independent of participant age, education level, and baseline HbA_1c_ [27]. In a post-hoc analysis, within the CGM group, the largest HbA_1c_ reductions were achieved for patients with the highest baseline HbA_1c_ levels (≥9.0%) [28]. Also, results from both the DIAMOND and GOLD studies showed that CGM use was associated with a significantly reduced time spent in – and episodes of – hypoglycaemia, particularly overnight [29,30].

##### CGM and time in range

Time in range (TIR) is the proportion of time that people with diabetes spend within the desired target glucose range (usually 70–180 mg/dL), and is an increasingly relevant metric of glycaemic control that provides more immediately actionable information than HbA_1c_ level [33]. Information on TIR, including time spent outside of range and variability, can only currently be obtained by using CGM [4]. Traditionally, HbA_1c_ has been the standard method for assessing glycaemic control, but it only gives the average over a 2–3-month period [25,34]. HbA_1c_ can be estimated using CGM and the Glucose Management Indicator (GMI) formula, formerly known as estimated HbA_1c _and calculated as GMI (%) = 3.31 + 0.02392 × [mean glucose in mg/dL] [35]. However, whether actual or estimated by GMI, HbA_1c _does not reflect intra- and inter-day glycaemic excursions that may lead to acute events (such as hypoglycaemia) or postprandial hyperglycaemia, which have been linked to both micro- and macrovascular complications [24]. Studies have shown a correlation between increased TIR and a reduction in diabetes complications [36–38]. As such, TIR is recommended as a new key metric of glycaemic control [25]. TIR measurements correlate with HbA_1c_ [37,39] and the two measures should be considered complementary in decision-making for diabetes management [33]. It is recommended that patients achieve >70% TIR, with <5% time below range (<70 mg/dL) and <25% time above range (>180 mg/dL) [25].

##### Practical information for PCPs

Patients need to be mindful of the potential for knocking off the CGM sensor, and that irritation from adhesives used to secure the device may occur. Some technical issues with software and connectivity may arise. From the PCP’s perspective, neither SMBG nor CGM is a replacement for keeping a clinical overview of the patient. Some CGM devices require calibration with blood glucose levels (Medtronic^® ^Guardian), necessitating the concurrent use of SMBG [19]. However, newer devices are factory-calibrated and do not require finger-stick calibrations (Freestyle Libre^®^), or calibration is optional (Dexcom^® ^G6) [21]. Although there is a good correlation between interstitial and blood glucose levels in the case of stable glucose levels, there is a time lag (approximately 5–7 min) between the blood glucose and interstitial glucose levels. This is most evident when the glucose levels are changing rapidly, e.g. around exercise and mealtimes [19,40]. Patients should be advised of this to avoid concern when checking CGM values against SMBG. They also should be advised to always check an SMBG when low blood glucose is suspected, and use SMBG to determine when hypoglycaemia has been adequately treated [40]. Most CGM devices use disposable sensors that last between 7 and 14 days [21]. One device has an implantable sensor (subcutaneous on upper arm) with a 90-day lifespan (Eversense^®^) [21,41].

Although there are many advantages with CGM, it must be noted that an accurate glucose reading is still required to determine the dose of basal-bolus insulin. Both HbA_1c_ and CGM sample measurements are helpful to see the full clinical picture. Although CGM can help patients to optimise their glycaemic management, psychosocial support may be required in addition to technical training [42]. CGM may not be suitable for everyone, potentially including patients who have high levels of anxiety, for example [43]. Furthermore, a greater understanding of the effect of diet on the postprandial blood glucose would allow for individualisation of appropriate insulin delivery when insulin:carbohydrate ratios are insufficient.

### Diabetes technologies for insulin administration

Of the insulin administration technologies available, PCPs are most likely to be involved in supporting patients in using insulin pens. Insulin pumps and closed-loop systems (integrated CGM plus an insulin pump) are increasingly being used, but generally only for patients with T1D. Nevertheless, it is important for PCPs to understand the clinical application of these new technologies. Table 2 provides a summary of devices for administering insulin.

**Table 2.** Attributes of diabetes technologies approved for insulin delivery [4,11,12,44–51].

	Insulin pens	Connected insulin pens	Insulin pumps
	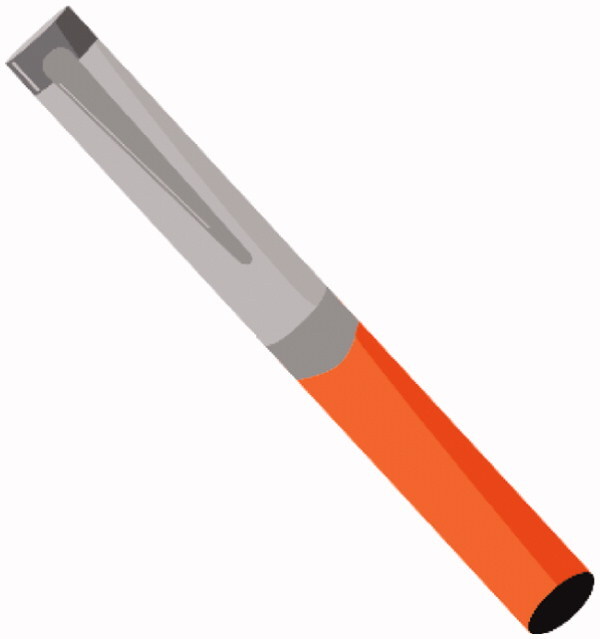	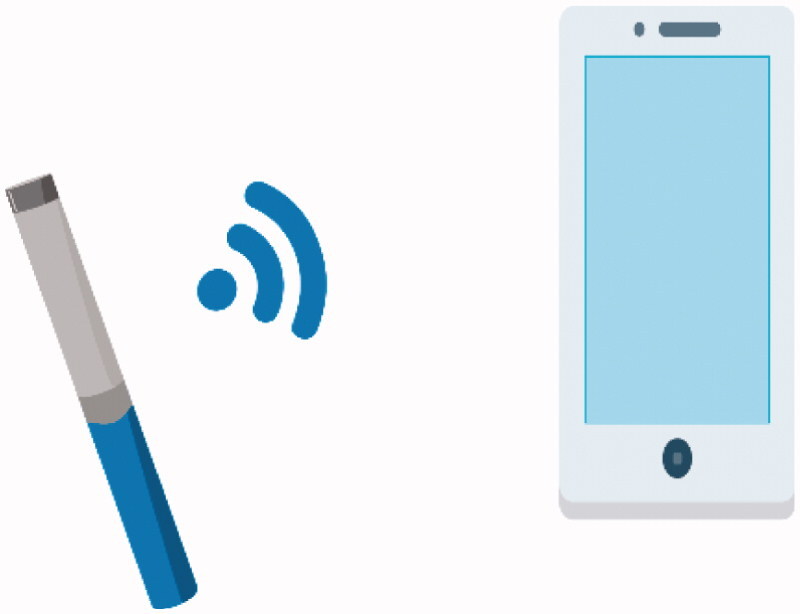	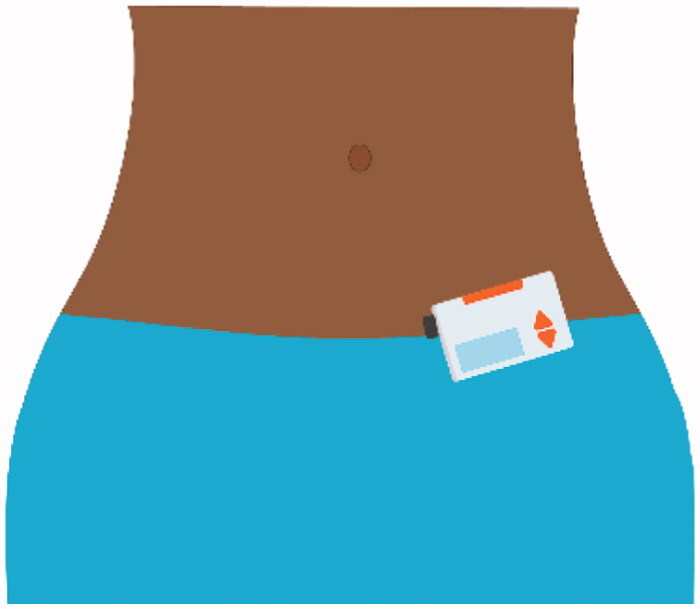
Example devices	Basaglar KwikPen^®^, Lantus/Toujoeo SoloStar^®^, Levemir FlexTouch^®^, Tresiba FlexTouch^®^	InPen^®^, NovoPen^® ^Echo Plus*	MiniMed^®^, Accu-Chek^®^, Omnipod^®^, Tandem^®^
Technology	Vial with insulin and syringe are combined in a single device; allow push button injections Disposable pens have a prefilled cartridge; reusable pens have replaceable insulin cartridges	Insulin pen can record amount and timing of each insulin dose; electronic display shows amount of insulin in pen, size of last dose, and time since last injection Able to wirelessly transmit information *via* Bluetooth to a dedicated mobile app	Wearable electromechanical pump: battery-operated motor, computerised control mechanism, insulin reservoir, and infusion set (s.c. cannula and tubing) Tubing-free pumps have a reservoir and integrated infusion set that adhere to skin Control panel or wireless controller to program basal and bolus insulin delivery A closed-loop system (“artificial pancreas”) that automatically adjusts insulin dose based on CGM is available
Software and applications	None	Smart phone app tracks insulin administered; make dosing recommendations; prepares reports for healthcare teams Integrated systems in development to connect insulin pen data with data from other diabetes technologies (such as CGM/SMBG systems) Most manufacturers provide apps and/or websites for integrating data across diabetes care	Different devices are supported by a range of software and apps, including: Wireless handset acts as a blood glucose monitor, bolus advisor, data manager, and remote control for the pump, linked *via* Bluetooth Bluetooth connections to remote control app on smartphone; allows bolus calculations and data sharing/review Most manufacturers provide apps and/or websites for integrating data across diabetes care
Advantages	Convenient Shorter, thinner needles reduce injection pain and risk of intramuscular injection versus longer needles Associated with improved adherence and lower hypoglycaemia risk versus syringes Flexible dosing; delivering insulin in increments of 0.5–2 units (good for children and those needing tight insulin control) Allow people with visual impairment or dexterity issues to dose accurately	Convenient Dosing accuracy; captures record of timing/amount of insulin administered Improved time in range Supports adherence/compliance including in people who have numeracy or cognitive issues Better support for people starting on insulin therapy or for whom hypoglycaemia is an ongoing issue	Convenient, efficient and flexible Able to vary basal insulin at different times of the day Easy and painless to adjust insulin when needed Supports adherence/compliance Associated with improved glycaemic control and clinical outcomes Suitable for adult and paediatric use
Disadvantages	More expensive than syringes/insulin vials Different types of insulin have different pens, limiting patient preferences Do not capture insulin dose or timing of injection	More expensive than disposable insulin pens Requires smartphone and internet connectivity (limitation for some people with diabetes)	High cost versus MDI Some are complex to use Risk of insulin errors due to pump failure, insulin infusion set blockage, infusion site problems, insulin stability issues, user error, or a combination of these Risk of serious complications (e.g. ketosis and DKA) if infusion set becomes dislodged or occluded; pump site becomes infected Potential for user issues with disliking pump, psychological impact (e.g. anxiety or depression)
Other considerations	Training in proper technique is a requisite to obtain full benefits	Education for HCPs and training for insulin users are required to realise the potential benefits of data captured	Age-appropriate structured continuous education of the entire family (and possibly also of kindergarten/school personnel) is key Cost is prohibitive if no insurance coverage

*Connected version not yet approved.

CGM: continuous glucose monitoring; DKA: diabetic ketoacidosis; HCP: healthcare professional; MDI: multiple daily injections; s.c.: subcutaneous; SMBG: self-monitored blood glucose.

#### Insulin pens

Insulin pen devices comprise an insulin cartridge and syringe combined in a single device for insulin administration [4,44]. The advantages of disposable insulin pens over vials/syringes include increased simplicity and convenience, as well as potential efficacy and safety improvements [44–46]. Disposable insulin pens do not capture data (although some reusable pens include a memory function, capturing dosages and timing). Thus, information on food intake, insulin dose, and timing of injection needs to be recorded in a diary. A lack of accurate record-keeping regarding insulin is a significant barrier to optimising glycaemic control for individuals using insulin pens [11], and this is now increasingly being addressed by the introduction of connected pens.

##### Smart pens

Connected (“smart”) insulin pens, which link to software applications (apps) that can be programmed to calculate insulin doses and provide downloadable data reports, have recently become available in the USA (11). They are able to store data on the date and time of injections and number of units administered, which can be downloaded using near-field connectivity and downloaded *via* Bluetooth to a centralised database on a computer-based data visualisation program; this allows both the insulin user and their healthcare teams to access accurate information on insulin administration and insulin injection patterns over time [47,48]. In a prospective observational study, switching to a connected pen resulted in significantly improved TIR, reduced time in hyperglycaemia, and reduced the number of missed bolus doses [47]. Advancing the technology further, a connected pen with a bolus calculator that can calculate the insulin dose based on the carbohydrates the patient will consume, plus the correction dose needed to get the glucose back to target, is now available [48]. The calculation is done by a smartphone app. The healthcare provider can download the data from the pen, along with CGM data, and this can aid in improving the management of the patient’s diabetes by informing adjustment of the insulin:carbohydrate ratio at each meal, the correction factor, and target range.

##### Practical considerations for PCPs

Insulin pens are often prescribed with a separate prescription for the pen needles. Patients should be made aware that the insulin pen is only active at room temperature, and pens that are “in-use” can be left at room temperature. Adequate time needs to be set aside in the clinic appointment to demonstrate how to use the insulin pen. Patients may incorrectly administer insulin by dialling down the pen without pushing the plunger, which results in confusion as to whether the dose was administered. Patients may forget if a dose was given and/or may mix up doses of different insulins (long vs. short acting). Connected pens can help to clarify this for the patient and PCP.

Technology can help to inform and guide the process of insulin initiation and titration. With the avoidance of hypoglycaemia in mind, basal insulin is usually started at a low dose (0.1–0.2 units/kg/day), after which dose titration is guided by SMBG to achieve a fasting plasma glucose target, usually in the range 80–130 mg/dL [8]. A gradual increase (1 unit per day or 2–4 units once or twice per week) is suggested, up to a maximum of 0.5 units/kg/day [52]. Beyond this, addition of mealtime insulin or (if not already prescribed) other classes of glucose-lowering medication is necessary. A mealtime insulin regimen should start with 2–4 units of rapid insulin at each meal, adjusted once or twice per week to maintain a 2-h post-meal glucose reading <180 mg/dL, or the next mealtime or bedtime reading 80–130 mg/dL. It should be emphasised that blood glucose values on their own, without information on the insulin dose and carbohydrate intake, is insufficient to making meaningful conclusions and modifications to insulin dosing.

There is one inhaled insulin currently available, used for mealtime boluses; it does not remove the need for subcutaneous injection if basal insulin is needed, however, and is contraindicated in patients with asthma or chronic lung disease [21].

#### Insulin pumps

Insulin pump therapy, also known as CSII, is an important and evolving form of insulin delivery; it is used mainly for people with T1D who are motivated to improve glycaemic control [12,50] but many people with T2D are now also using pump therapy. It involves patients wearing a portable electromechanical pump that infuses rapid-acting insulin at pre-selected basal rates throughout the day. The mealtime dose is given based on the food intake of carbohydrates, as well as the current glucose measurement and the insulin on board from a meal [51]. Results from a systematic review and meta-analysis concluded that pump therapy has the potential to improve glycaemic control with a reduction in hypoglycaemia [53]. A sizeable minority of people with T2D are now using simplified patch pumps [54], and this may represent the future of insulin delivery for T2D once suitable automation is readily available.

##### Integrated pump systems

Recent advances in pump technology include the development of sensor-augmented pumps, integrated with a real-time CGM, allowing hybrid closed-loop systems to be developed (the so-called “artificial pancreas”). Automated insulin delivery systems increase and decrease insulin delivery based on sensor-derived glucose levels to approximate physiologic insulin delivery. These systems consist of three components: an insulin pump, a CGM sensor, and an algorithm that determines insulin delivery. Insulin delivery can be stopped, increased, or decreased based on sensor glucose values [49]. In 2017, the FDA approved the first automated insulin delivery system that automatically adjusts insulin delivery every 5 min based on the sensor glucose.

## Discussion

### Best practice for use of technology for primary care management of people with diabetes

The ADA guideline recommendations on the use of technologies in people with diabetes are summarised in Tables 3 and 4, highlighting key actions for healthcare providers [4]. The guiding principle is that use of technology should be individualised based on a patient’s needs, desires, skill level, and availability of devices [4]. Healthcare teams should support people with diabetes to choose the device/program that is best suited to their needs and skills, and support its use through ongoing education and training.

**Table 3.** Summary of ADA standards of medical care in diabetes 2020: glucose monitoring (focus on people with type 2 diabetes) [4].

Technology	Recommendation on clinical use	Actions for HCPs
SMBG	**People on intensive insulin regimens** Most people using intensive insulin regimens (MDI or insulin pump therapy) should be encouraged to assess glucose levels using SMBG (and/or CGM) Prior to meals/snacks, at bedtime, prior to exercise, when suspect low glucose, after treating low blood glucose until normoglycaemic, prior to and while performing critical tasks (e.g. driving) **People on less-frequent insulin regimens** SMBG may help to guide treatment decisions and/or self-management for patients taking less-frequent insulin injections, when prescribed as part of a diabetes self-management education and support **People on non-insulin regimens** SMBG may be helpful in patients on non-insulin therapies when altering diet, physical activity, and/or medications (particularly medications that can cause hypoglycaemia), in conjunction with a treatment adjustment program Although SMBG has not shown clinically significant reductions in HbA_1c_	When prescribing SMBG, ensure that patients receive ongoing instruction and regular evaluation of technique, results, and their ability to use data from SMBG to adjust therapy Be aware of medications, e.g. high-dose vitamin C, acetaminophen, and other factors, e.g. hypoxaemia, that can interfere with glucose meter accuracy, and provide clinical management Be aware of the differences in accuracy among glucose meters – only US FDA-approved meters should be used with unexpired strips, purchased from a pharmacy or licenced distributor
CGM	**Adults with T2D** Real-time and intermittently scanned CGM (when used properly) in conjunction with insulin therapy are useful tools to lower A1C and/or reduce hypoglycaemia in adults with T2D who are not meeting glycaemic targets **Frequency of use** Real-time CGM devices should be used throughout the day for maximal benefit Intermittently scanned CGM devices should be scanned frequently, at a minimum once every 8 h **Use of blinded CGM data** Blinded CGM data, when coupled with diabetes self-management education and medication dose adjustment, can be helpful in identifying and correcting patterns of hyper- and hypoglycaemia in people with T1D and T2D (as long as the data can be accessed/downloaded for office use)	When prescribing CGM devices, robust diabetes education, training, and support are required for optimal CGM device implementation and ongoing use People using CGM devices need to have the ability to perform SMBG in order to calibrate their monitor and/or verify readings if discordant from their symptoms People who have been using CGM should have continued access across third-party payers

ADA: American Diabetes Association; CGM: continuous glucose monitoring; FDA: Food and Drug Administration; HbA_1c_: glycated haemoglobin; HCP: healthcare professional; MDI: multiple daily injections; SMBG: self-monitored blood glucose; T1D: type 1 diabetes; T2D: type 2 diabetes.

**Table 4.** Summary of ADA standards of medical care in diabetes 2020: insulin delivery devices [4].

Technology	Recommendation on clinical use	Actions for HCPs
Insulin syringes and pens	**People with diabetes on insulin regimens** Insulin syringes or insulin pens may be used for insulin delivery with consideration of patient preference, insulin type and dosing regimen, cost, and self-management capabilities **People with dexterity issues or visual impairment** Insulin pens or insulin injection aids may be considered **Smart pens** Smart pens may be useful for some patients to help with dose capture and dosing recommendations **Insulin dose calculators/decision support systems** US FDA-approved insulin dose calculators/decision support systems may be helpful for titrating insulin doses	Patients using insulin should have an examination of insulin injection/infusion sites on a routine basis – at least annually and if there are clinical issues related to insulin delivery Competent patients using diabetes devices should be allowed to use them in an inpatient setting when proper supervision is available
Insulin pumps	**People with T1D (could also be appropriate for hard-to-control T2D)** Insulin pumps may be considered as an option for all adults, children, and adolescents with T1D who are able to safely manage the device	Individuals with diabetes who have been successfully using CSII should have continued access across third-party payers
Combined insulin pump and sensor systems	**People with T1D (adults and children; could also be appropriate for hard-to-control T2D)** Sensor-augmented pump therapy with automatic low glucose suspend may be considered to prevent/mitigate episodes of hypoglycaemia Automated insulin delivery systems should be considered in adults with T1D who have the skills to use them to improve time in range, and reduce A1C/hypoglycaemia; may also be useful to improve glycaemia in children	Individual patients may be using systems not approved by the US FDA (e.g. DIY closed-loop systems) Providers cannot prescribe these systems but can provide safety information, troubleshooting, or backup advice for the individual devices to enhance patient safety

ADA: American Diabetes Association; CSII: continuous subcutaneous insulin infusion; DIY: do it yourself; FDA: Food and Drug Administration; HCP: healthcare professional; T1D: type 1 diabetes; T2D: type 2 diabetes; US: United States.

Training programs/tutorials on new technology are essential for both the healthcare teams and the people using the technology. Non-profit websites exist to offer advice for providers and patients to determine the suitability of various options (for example DiabetesWise.org).

With direct-to-consumer marketing, patients may increasingly ask their PCP for diabetes technology, putting the onus on PCPs to be up to date on these technologies. Therefore, there is some need for PCPs to be “digitally savvy” and keep abreast of technological advances in diabetes care. The challenge of insurance coverage/payment for expensive new technologies is likely to drive further health inequalities and financial pressure on people living with diabetes; documenting the type of diabetes, number of injections, and number of glucose checks is needed to support an application for CGM. Therefore, decisions about integrating technology into care plans need to be made in consideration of the social context (including financial barriers), technical capabilities, and support networks for each person with diabetes.

### Software and applications

Integration of diabetes technology with the healthcare provider and patient is a key factor that contributes to the success of these new technologies. Various software and apps are available to support healthcare professionals and patients, which can aid conversations within clinics and support clinical decisions. Smart pens have applications or platforms that allow PCPs and patients to visualise the data from the device. There are also platforms (e.g. Glooko, Tidepool) that can integrate data from both smart devices for insulin administration and monitoring, to foster conversations between the provider and patient about glucose trends, bolus timing, missed boluses, etc. FDA-approved insulin dose calculators/decision support systems may be helpful for titrating insulin doses.

The wide variety of software and apps, and the variability in their features may make it difficult for patients to select the most appropriate app [55]. Individual healthcare providers and patients may have to try different apps to find ones that work for them. There are important distinctions between FDA-approved software/apps and those that are not, as well as issues with data privacy [4,56]. Challenges with apps that need to be addressed include: inadequate evidence on app accuracy and clinical validity; lack of training provision; poor interoperability and standardisation; and insufficient data security [56]. There is currently a lack of official guidance from professional organisations, but a joint ADA/European Association for the Study of Diabetes working group has recommended actions to overcome these shortcomings [56].

### Future perspectives

Diabetes technologies are constantly evolving. They include implantable glucose sensors and drug-delivery systems, enhanced automated closed-loop systems, and miniaturised non-invasive glucose monitoring systems [41]. As an example, a study of automatic titration of insulin dose to improve TIR in patients with T2D managed by CGM was recently published [57]. These technologies are not yet routinely used in patients with T2D managed in primary care, but this will likely change in the future.

Further development of novel sensors, capture of “big data,” and use of artificial intelligence can be expected, which are likely to provide advances for preventing, monitoring, and treating diabetes [58]. However, the use of a new technology does not guarantee improved care; the underlying principles of diabetes care remain unchanged [4]. Education and counselling from healthcare providers, including Certified Diabetes Care and Educational Specialists, are crucial for people with diabetes who are not at goal.

## Conclusion

Advancements in diabetes technologies continue to improve the accuracy of glucose monitoring and regulation, aiding clinical decision making and individualised management in the primary care setting. The wide variety of devices and technology available for self-management will help patients improve their clinical outcomes, decrease disease burden, and achieve the quality of life they desire.

## Supplementary Material

Supplemental Material
