# Supplementary material for: Leveraging advances in diabetes technologies in primary care: a narrative review
Source: Ann Med. 2021 Jun 29;53(1):805–16. doi: 10.1080/07853890.2021.1931427 (PMC8245065; doi:10.1080/07853890.2021.1931427)
Supplement: Supplemental Material [file IANN_A_1931427_SM1442.pdf]

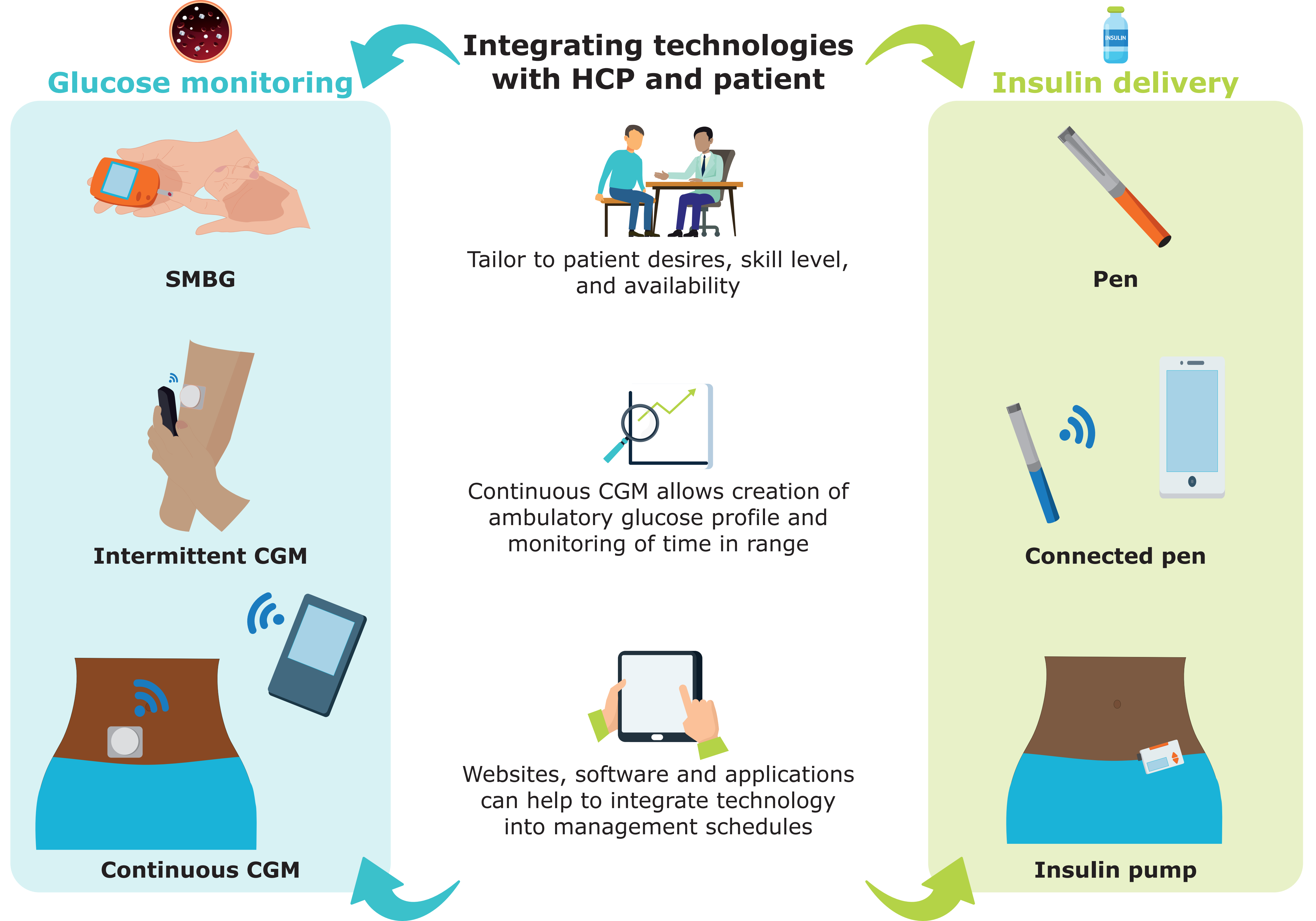
Integrating technologies with HCP and patient
Glucose monitoring
INSULIN
Insulin delivery
SMBG
Tailor to patient desires, skill level, and availability
Pen
Intermittent CGM
Continuous CGM allows creation of ambulatory glucose profile and monitoring of time in range
Connected pen
Websites, software and applications can help to integrate technology into management schedules
Continuous CGM
Insulin pump
